# Risk Stratification of Adverse Outcomes After Heart Transplantation with the 2022 Definition of Pulmonary Hypertension

**DOI:** 10.3390/jcdd13060227

**Published:** 2026-05-27

**Authors:** Mattia Corianò, Nicola Pradegan, Francesco Putortì, Arianna Calonaci, Andrea Golfetto, Vincenzo Tarzia, Annalisa Angelini, Chiara Tessari, Marny Fedrigo, Giuseppe Toscano, Gino Gerosa, Francesco Tona

**Affiliations:** 1 Cardiology Unit, Department of Cardiac, Thoracic, Vascular Sciences and Public Health, University of Padua, 35100 Padua, Italy; mattia.coriano@studenti.unipd.it (M.C.);; 2 Cardiac Surgery Unit, Department of Cardiac, Thoracic, Vascular Sciences and Public Health, University of Padua, 35100 Padua, Italy; 3 Pathology and Pathological Anatomy Unit, Department of Cardiac, Thoracic, Vascular Sciences and Public Health, University of Padova, 35100 Padova, Italy; 4 Cardiovascular Pathology, Department of Integrated Diagnostic Services, Hospital-University of Padua, 35128 Padua, Italy

**Keywords:** heart transplantation, pulmonary hypertension, pulmonary resistance

## Abstract

Pulmonary hypertension (PH) is associated with adverse outcomes after heart transplantation (HT). In 2022, the European Society of Cardiology introduced lower thresholds for pulmonary vascular resistance (PVR) to distinguish isolated post-capillary PH (IpcPH) and combined post-capillary PH (CpcPH). We conducted a single-center retrospective study on 357 patients who underwent HT between 1985 and 2020 and had right heart catheterization prior to transplant, investigating the ability of the new PVR threshold to predict one-year mortality. Overall, 65 patients had no PH, 84 had IpcPH, and 208 had CpcPH. One-year survival was higher in patients without PH and similar between IpcPH and CpcPH (*p* = 0.04). Reclassification under the 2022 guidelines did not improve risk prediction. Only mean pulmonary artery pressure (mPAP) >20 mmHg independently predicted 1-year mortality. In conclusion, elevated mPAP, rather than PVR, was associated with post-transplant outcomes. This finding opens up the possibility of rethinking indication for reversibility tests and mechanical circulatory support in HT recipients.

## 1. Background

Pulmonary hypertension (PH) in heart transplant (HT) recipients has been associated with higher rates of graft failure and mortality at both 30 days and 1 year post-transplant compared to normal pre-transplant values [1,2,3]. Patients with systolic pulmonary arterial pressure (sPAP) ≥50 mmHg and pulmonary vascular resistance (PVR) ≥3 WU are considered at higher risk and current recommendations suggest performing a vasodilator test to evaluate candidacy [4]. In 2022, the European Society of Cardiology released an updated classification of PH, defining isolated post capillary PH (IpcPH) as a mean PAP (mPAP) ≥20 mmHg and PVR ≤2 WU, and combined pcPH (CpcPH) as mPAP ≥20 mmHg and PVR >2 WU [5].

To date, the prognostic value of these new thresholds has not been evaluated in a prospective cohort of patients awaiting HT. Therefore, the aim of this study was to investigate whether the updated PH classification affects risk stratification in this population. Specifically, by applying the new PVR cut-off values, we sought to determine whether reclassifying patients into IpcPH and CpcPH according to 2022 guidelines would provide additional prognostic insights on post-HT outcome.

## 2. Methods

In this single-center, retrospective, observational cohort study, data from the historical cohort of patients who received HT at our institution from January 1985 to December 2020 were screened. All patients who underwent right heart catheterization (RHC) before HT were included. Exclusion criteria were age <18; RHC performed more than one year before HT; incomplete RHC data; and type 1 PH according to 2022 guidelines [5]. RHC data were obtained by reviewing medical records. The following variables were collected: right atrial pressure (RAP), mPAP, sPAP, diastolic PAP, pulmonary capillary wedge pressure (PCWP), PVR, cardiac index (CI), stroke volume (SV), transpulmonary pressure gradient (TPG), and pulmonary arterial compliance (PAC).

**Figure 1:**
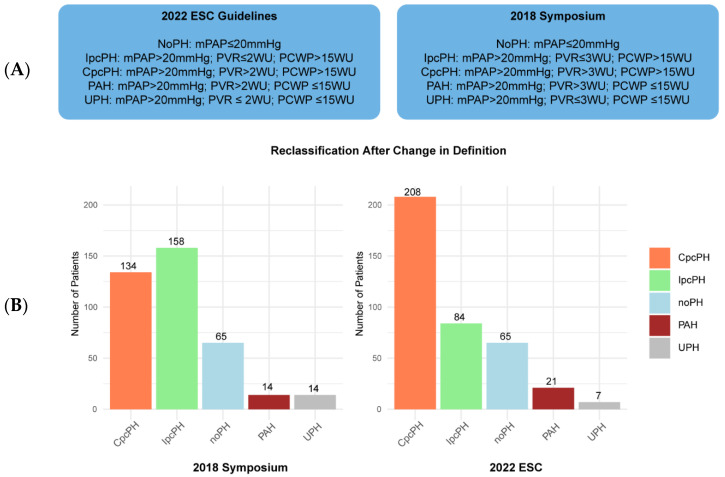
(**A**) Definition of pulmonary hypertension according to the 2018 World Symposium and 2022 European Society of Cardiology/European Respiratory Society guidelines. (**B**) Reclassification of patients according to the 2022 definition. Abbreviations: CpcPH, combined post-capillary pulmonary hypertension; IpcPH, isolated post-capillary pulmonary hypertension; noPH, no pulmonary hypertension; PAH, pulmonary arterial hypertension; UPH, undefined pulmonary hypertension.

Baseline data on demographics and medical history were also collected. The cohort was grouped into three groups according to the 2022 definition: absence of PH (NoPH), IpcPH, and CpcPH [5]. The cohort was also grouped according to the previous 2018 definition of PH (Figure 1A) and the number of reclassified cases was evaluated [6]. The primary endpoint was a composite of all-cause death or retransplant at 1 year after HT. Follow-up data were obtained by reviewing medical records.

Continuous and categorical variables were compared across the three groups using Kruskal–Wallis or χ^2^ test, respectively. The survival rate free from the primary endpoint was estimated using the Kaplan–Meier method, and differences between groups were evaluated using the Log-rank test. The Cox proportional hazards regression model was used to calculate hazard ratios for mortality for RHC variables.

To further investigate the prognostic performance of the updated pulmonary hypertension classification, a risk stratification analysis for the 5-year composite endpoint of all-cause death or retransplant after HT was performed. Cox proportional hazards regression models were constructed using a baseline clinical model including recipient age, sex, heart failure etiology, and donor age. The prognostic contribution of the previous (2018 World Symposium) and updated (2022 ESC/ERS) PH classifications was then assessed by sequentially adding each classification to the baseline model. Model discrimination was evaluated using Harrell’s concordance statistic (C-statistic) [7], and the predictive performance of the two classifications was compared using the *compareC* test function for right censored survival data, as proposed by Kang et al. [8]. A two-sided significance level of α = 0.05 was considered appropriate to indicate statistical significance. Statistical analyses were performed with R (Version 4.3.2).

This study was conducted in accordance with the principles of the Declaration of Helsinki, the Declaration of Istanbul, and the ethical standards of the International Society for Heart and Lung Transplantation, and it was approved by the Ethics Committee for Clinical Trials of the host institution (“Comitato Etico Territoriale Area Centro—Est Veneto”; Approval Code: 343n/AO/23; Approval Date: 1 June 2023). The data that support the findings of this study are available from the corresponding author upon reasonable request.

## 3. Results

The final cohort consisted of 357 patients. Most of the recipients (81%) and donors (63%) were male, with median ages of 55 (44–60) and 32 (20–46) years, respectively. The two main causes of HT were dilated cardiomyopathy (42%) and ischemic heart disease (40%).

**Figure 2:**
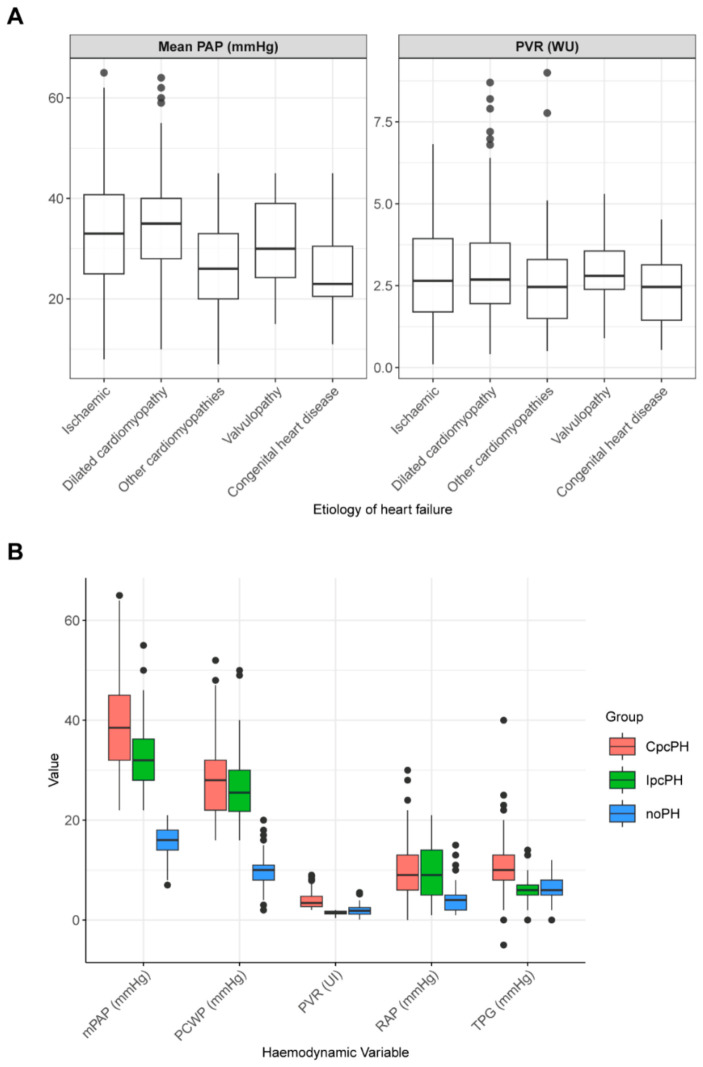
(**A**) Mean pulmonary arterial pressure (**left**) and pulmonary vascular resistance (**right**) according to heart failure etiology. “Other cardiomyopathies” includes arrhythmogenic, hypertrophic, and restrictive cardiomyopathies. (**B**) Hemodynamic characteristics of heart transplant candidates classified according to the 2022 definition. Abbreviations: CpcPH, combined post-capillary pulmonary hypertension; IpcPH, isolated post-capillary pulmonary hypertension; mPAP, mean pulmonary arterial pressure; noPH, no pulmonary hypertension; PCWP, pulmonary capillary wedge pressure; PVR, pulmonary vascular resistance; RAP, right atrial pressure; TPG, transpulmonary gradient.

According to the 2022 definition, 65 (17%) patients had noPH, while 84 (23%) had IpcPH and 208 (54%) had CpcPH. Overall, mPAP, PCWP and PVR were 34 mmHg (24–40), 25 mmHg (19–30) and 2.6 WU (1.7–3.8), respectively. Compared to the 2018 definition, 74 (47%) patients with IpcPH were reclassified as having CpcPH with the updated definition (Figure 1B). mPAP differed significantly across aetiologic groups (ischemic vs. dilated cardiomyopathy vs. other; Kruskal–Wallis χ^2^ = 17.9, *p* = 0.0001), while PVR was comparable among groups and did not significantly differ according to heart failure etiology (Kruskal–Wallis χ^2^ = 3.6, *p* = 0.16), as shown in Figure 2A.

After classifying patients according to the 2022 definition, the three groups did not differ regarding age, sex, indication for HT, donor characteristics and mismatch of BMI and predicted heart mass. mPAP was higher in CpcPH (39 mmHg vs. 16 mmHg and 32 mmHg for noPH and IpcPH, respectively, *p* < 0.001), while PCWP was higher than noPH but similar to IpcPH (28 mmHg vs. 10 mmHg and 26 mmHg for noPH and IpcPH, respectively, *p* < 0.001). On the contrary, PVR was lower in IpcPH compared to the other groups (1.49 WU vs. 1.83 WU and 3.4 WU, respectively, *p* < 0.001).

The noPH group exhibited higher cardiac index (2.28 L/min/m^2^ vs. 2.12 L/min/m^2^ and 2.00 L/min/m^2^ for IpcPH and CpcPH, respectively [*p* < 0.001]). Moreover, right atrial pressure was significantly lower in noPH than in IpcPH and CpcPH (4 mmHg vs. 9 mmHg and 9 mmHg, *p* < 0.001), while pulmonary arterial compliance was significantly higher (3.0 mL/mmHg vs. 2.0 mL/mmHg and 1.60 mL/mmHg, *p* = 0.002). On the contrary, TPG was higher in CpcPH compared to noPH and IpcPH (10 mmHg vs. 6 mmHg and 6 mmHg, *p* < 0.001). No differences in mean arterial pressure, heart rate and stroke volume were noted (Figure 2B).

**Figure 3:**
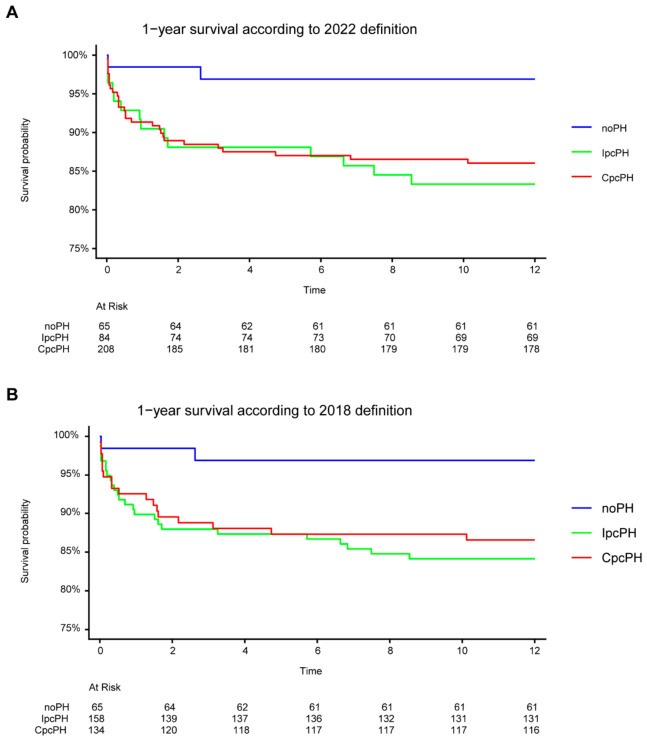
(**A**) One-year survival according to the 2022 pulmonary hypertension definition. (**B**) One-year survival according to the 2018 pulmonary hypertension definition. Abbreviations: CpcPH, combined post-capillary pulmonary hypertension; IpcPH, isolated post-capillary pulmonary hypertension; noPH, no pulmonary hypertension.

After 12 months of follow-up, the primary endpoint was observed in 48 patients. When classifying patients according to the 2022 definition, 1-year survival was higher in noPH but similar between IpcPH and CpcPH (97% [0.93–0.99] vs. 83% [0.76–0.92] and 86% [0.81–0.91], respectively, LogRank *p* = 0.04) (Figure 3A). Similar differences in 1-year survival were observed when classifying patients according to the 2018 definition (Figure 3B). After adjusting for confounding variables, only mPAP > 20 mmHg was associated with 1-year mortality (HR 4.31 [1.04–17.9], *p* = 0.044), while PCWP, PVR > 2 WU and >3 WU did not predict the primary endpoint (*p* = 0.511, *p* = 0.580, *p* = 0.478, respectively) (Table 1).

In the 5-year risk stratification analysis, the baseline clinical Cox model showed modest discriminative ability (Harrell’s C-statistic 0.571). The addition of the previous PH classification marginally improved model discrimination (C-statistic 0.595), while the updated PH classification yielded a similar performance (C-statistic 0.591). Direct comparison between the two prognostic models demonstrated no significant difference in discrimination for the 5-year endpoint (ΔC-statistic −0.001, *p* = 0.84).

## 4. Discussion

In this study, we investigated the impact of the updated 2022 ESC/ERS pulmonary hypertension definition on post-transplant outcomes and risk stratification in patients undergoing heart transplantation. We found that implementation of the updated definition substantially increased the prevalence of CpcPH, with nearly half of patients previously classified as IpcPH being reclassified. Although patients with PH had worse outcomes compared with those without PH, survival remained similar between IpcPH and CpcPH irrespective of the PVR threshold applied. Moreover, mPAP >20 mmHg, rather than revised PVR thresholds, independently predicted adverse outcomes, while incorporation of the updated classification into a multivariable prognostic model did not improve discrimination for 5-year adverse outcomes compared with the previous definition.

Elevated PVR has historically been considered a major determinant of adverse outcomes after HT because of its association with right ventricular failure and post-transplant mortality [9]. Previous studies and registry analyses demonstrated an association between pre-transplant pulmonary hypertension and reduced survival after HT, leading to incorporation of PVR thresholds into transplant candidate evaluation algorithms [1,2,10]. Contemporary ISHLT guidelines recommend vasodilator challenge in transplant candidates with PASP ≥50 mmHg accompanied by either TPG ≥15 mmHg or PVR >3 WU [4]. Persistent elevation of PVR >5 WU or TPG >16–20 mmHg despite reversibility testing is considered a relative contraindication to isolated HT and may prompt consideration of left ventricular assistant device support as a bridge to candidacy [11].

Lowering the PVR threshold from >3 WU to >2 WU was introduced following evidence suggesting that even mildly increased pulmonary vascular resistance may be associated with adverse outcomes in cardiovascular disease [10,12]. The revised PH definition and recent clinical recommendations emphasized that these updated criteria should be interpreted within the broader clinical and pathophysiological context rather than as isolated hemodynamic cut-offs [5]. In our cohort, implementation of the revised definition substantially increased the prevalence of CpcPH. However, this reclassification did not translate into improved prognostic discrimination. These findings suggest that expanding the hemodynamic definition of CpcPH may increase sensitivity for identifying pulmonary vascular abnormalities without necessarily improving risk stratification among HT candidates.

From a clinical perspective, our findings may have implications for the evaluation of HT candidates. The ongoing TEAM-HF trial is testing the hypothesis that elevated mPAP may represent an objective marker to identify patients with advanced heart failure who could benefit from earlier implementation of mechanical circulatory support [13]. In line with this concept, our findings suggest that mPAP, rather than revised PVR thresholds, may represent the hemodynamic parameter most strongly associated with adverse outcomes after HT, raising the possibility that earlier intervention strategies could improve patient prognosis. Although hypothesis-generating, these observations suggest that pulmonary pressure burden may deserve greater attention during pre-transplant assessment and should be further explored in prospective studies.

Several limitations should be acknowledged. First, the retrospective single-center design may have introduced selection bias and may limit the generalizability of our findings. Second, the relatively small number of outcome events resulted in wide confidence intervals and limited statistical power, particularly for subgroup analyses. Third, the study spans more than three decades (1985–2020), during which transplant selection criteria, perioperative management, and immunosuppressive strategies substantially evolved. To address this issue, we performed an exploratory sensitivity analysis accounting for secular trends by stratifying patients according to transplant era; however, subdivision into temporal groups substantially reduced sample size and event numbers, limiting the ability to detect significant differences. Fourth, the available data predominantly focused on survival outcomes, whereas information regarding other post-transplant adverse events was limited. Finally, residual confounding due to variables not included in the multivariable analyses cannot be excluded.

## 5. Conclusions

In this study, the use of the updated 2022 ESC/ERS pulmonary hypertension definition substantially increased the prevalence of CpcPH among HT candidates but did not improve post-transplant risk stratification compared with the previous classification. Elevated mPAP, rather than revised PVR thresholds, was associated with adverse outcomes after HT. Further prospective studies are needed to determine whether earlier intervention in patients with isolated mPAP elevation may improve post-transplant outcomes.

## Figures and Tables

**Table 1:** Haemodynamic predictors of 1-year adverse outcomes after heart transplantation.

Variable	HR (95% CI)	*p*-Value
mPAP > 20 mmHg	4.31 (1.04–17.9)	0.044
PVR > 2 WU	1.07 (0.53–2.14)	0.580
PVR > 3 WU	1.08 (0.57–2.02)	0.478
PCWP > 15 mmHg	1.89 (0.35–10.3)	0.511
RAP, mmHg	1.03 (0.97–1.09)	0.323
TPG, mmHg	1.02 (0.98–1.06)	0.300

CI, confidence interval; HR, hazard ratio; mPAP, mean pulmonary arterial pressure; PCWP, pulmonary capillary wedge pressure; PVR, pulmonary vascular resistance; RAP, right atrial pressure; TPG, transpulmonary gradient.

## Data Availability

Data are available from the corresponding author upon reasonable request.

## Abbreviations

BMI	Body Mass Index
CI	Cardiac Index
CpcPH	Combined Post-Capillary Pulmonary Hypertension
HR	Hazard Ratio
HT	Heart Transplantation
IpcPH	Isolated Post-Capillary Pulmonary Hypertension
mPAP	Mean Pulmonary Arterial Pressure
MCS	Mechanical Circulatory Support
NRI	Net Reclassification Improvement
PAC	Pulmonary Arterial Compliance
PCWP	Pulmonary Capillary Wedge Pressure
PH	Pulmonary Hypertension
PVR	Pulmonary Vascular Resistance
RAP	Right Atrial Pressure
RHC	Right Heart Catheterization
sPAP	Systolic Pulmonary Arterial Pressure
SV	Stroke Volume
TPG	Transpulmonary Pressure Gradient
WU	Wood Units
